# The economic burden of subjective cognitive decline, mild cognitive impairment and Alzheimer's dementia: excess costs and associated clinical and risk factors

**DOI:** 10.1186/s13195-025-01785-9

**Published:** 2025-06-26

**Authors:** Eva Gläser, Ingo Kilimann, Moritz Platen, Wolfgang Hoffmann, Frederic Brosseron, Katharina Buerger, Marie Coenjaerts, Emrah Düzel, Michael Ewers, Klaus Fliessbach, Ingo Frommann, Maria Gemenetzi, Wenzel Glanz, Julian Hellmann-Regen, Enise I. Incesoy, Daniel Janowitz, Frank Jessen, Oliver Peters, Josef Priller, Alfredo Ramirez, Anja Schneider, Annika Spottke, Eike Jakob Spruth, Stefan Teipel, Michael Wagner, Bernhard Michalowsky

**Affiliations:** 1 German Center for Neurodegenerative Diseases (DZNE) Rostock/Greifswald, Site Greifswald, Ellernholzstraße 1-2, Greifswald, 17489 Germany; 2 German Center for Neurodegenerative Diseases (DZNE) Rostock/Greifswald, Gehlsheimer Str. 20, Rostock, 18147 Germany; 3 Department of Psychosomatic Medicine, Rostock University Medical Center, Gehlsheimer Str. 20, Rostock, 18147 Germany; 4 Institute for Community Medicine, Section Healthcare Epidemiology and Community Health, University Medicine Greifswald, Ellerholzstraße 1-2, Greifswald, 17489 Germany; 5 German Center for Neurodegenerative Diseases (DZNE), Venusberg-Campus 1, Bonn, 53127 Germany; 6 German Center for Neurodegenerative Diseases (DZNE, Munich), Feodor-Lynen-Strasse 17, Munich, 81377 Germany; 7 Institute for Stroke and Dementia Research (ISD), University Hospital, LMU Munich, Feodor-Lynen-Strasse 17, Munich, 81377 Germany; 8 German Center for Neurodegenerative Diseases (DZNE), Leipziger Str. 44, Magdeburg, 39120 Germany; 9 Institute of Cognitive Neurology and Dementia Research (IKND), Otto-von-Guericke University, Leipziger Str. 44, Magdeburg, 39120 Germany; 10 Department of Old Age Psychiatry and Cognitive Disorders, University Hospital Bonn and University of Bonn, Venusberg-Campus 1, Bonn, 53127 Germany; 11 German Center for Neurodegenerative Diseases (DZNE), Charitéplatz 1, Berlin, 10117 Germany; 12 Department of Psychiatry and Psychotherapy, Charité, Charitéplatz 1, Berlin, 10117 Germany; 13 Department of Psychiatry and Neurosciences, Charité Universitätsmedizin Berlin, Charitéplatz 1, Berlin, 10117 Germany; 14 ECRC Experimental and Clinical Research Center, Charité – Universitätsmedizin Berlin, Charitéplatz 1, Berlin, 10117 Germany; 15 Department of Psychiatry, Medical Faculty, University of Cologne, Kerpener Strasse 62, Cologne, 50924 Germany; 16 Excellence Cluster on Cellular Stress Responses in Aging-Associated Diseases (CECAD), University of Cologne, Joseph-Stelzmann-Strasse 26, Köln, 50931 Germany; 17 Institute of Psychiatry and Psychotherapy, Charité – Universitätsmedizin Berlin, Hindenburgdamm 30, Berlin, 12203 Germany; 18 University of Edinburgh and UK DRI, 49 Little France Crescent, Edinburgh, EH16 4SB UK; 19 Department of Psychiatry and Psychotherapy, School of Medicine and Health, Technical University of Munich, and German Center for Mental Health (DZPG), , Ismaninger Str. 22, Munich, 81675 Germany; 20 Department of Psychiatry and Psychotherapy, Division of Neurogenetics and Molecular Psychiatry, Faculty of Medicine and University Hospital Cologne, University of Cologne, Kerpener Str. 62, Cologne, 50931 Germany; 21 Department of Psychiatry & Glenn Biggs Institute for Alzheimer’s and Neurodegenerative Diseases, 8300 Floyd Curl Drive, San Antonio, TX 78229 USA; 22 Department of Neurology, University of Bonn, Venusberg-Campus 1, Bonn, 53127 Germany

**Keywords:** Dementia, Cognition, Subjective cognitive decline, Mild cognitive impairment, Alzheimer’s disease, Apolipoprotein E, Economics, Utilization, Cost

## Abstract

**Background:**

With the availability of first disease-modifying treatments, evidence on costs across the entire Alzheimer's Continuum, especially for early disease stages, becomes increasingly important to inform healthcare planning, resource allocation, and policy decisions. This study assessed costs and cost-associated factors in patients with subjective cognitive decline (SCD), mild cognitive impairment (MCI) and Alzheimer's Disease (AD) dementia compared to healthy controls.

**Methods:**

The German DELCODE cohort study assessed clinical data, healthcare resource use, and informal care provision. Costs were calculated from payer and societal perspectives using standardized unit costs, and multivariate regression analyses identified cost-associated factors.

**Results:**

From a payer perspective, costs were elevated by 26% for SCD (adjusted mean 5,976€ [95%CI 4,598-7,355€]), 85% for MCI (8,795€ [6,200-11,391€]) and 36% for AD (6,454€ [2,796-10,111€]) compared to controls (4,754€ [3,586-5,922€]). Societal costs were elevated by 52% for SCD (adjusted mean 8,377€ [95%CI 6,009-10,746€]), 170% for MCI (14,886€ [9,524-20,248€]) and 307% for AD (22,481€ [9,994-34,969€]) compared to controls (5,522€ [3,814-7,230€]). APOE e4 negative patients showed higher costs compared to APOE e4 positive patients. Hypertension was associated with higher costs.

**Conclusions:**

Healthcare costs are already elevated in early subjective and objective cognitive impairment, driven by formal and informal care. The study emphasizes the importance of early interventions to reduce the economic burden and delay progression.

**Supplementary Information:**

The online version contains supplementary material available at 10.1186/s13195-025-01785-9.

## Background

Dementia represents an increasing challenge for healthcare systems and societies from a medical, societal and economic perspective. In 2019, almost 58 million people were estimated to be living with dementia. This number is expected to increase to over 150 million people by 2050 [1]. The associated costs were estimated at USD 1,313 billion in 2019 [2]. Dementia does not only cause direct medical costs, posing a burden on care and social systems, but also significant informal costs for family caregivers [2].

Alzheimer's disease (AD) causes the most prevalent form of dementia and covers a long continuum of disease stages. The causes of AD are not fully known, even though several disease mechanisms and risk factors have been identified [3]. The APOE e4 allele is one of the most potent genetic risk factors for sporadic AD [4]. It has been linked with amyloid-ß (Aß) plaque aggregation, tau neurofibrillary degeneration, microglia and astrocyte responses, and blood-brain barrier disruptions, thus promoting neurodegeneration [5]. Fourteen modifiable risk factors have been stated to be associated with dementia [6], offering perspectives for preventive interventions.

Pathophysiological changes in Alzheimer's disease (AD) begin years before the onset of clinical symptoms or measurable cognitive deficits [7]. New AD definitions reflect this early disease process, incorporating biomarker evidence to identify asymptomatic or preclinical stages [7]. Individuals in asymptomatic at-risk stages for AD are essential as a target population for early interventions to prevent or delay cognitive decline and, thus, the onset of dementia symptoms.

In this study, however, we focus on clinical at-risk stages defined by early cognitive symptoms rather than biomarker presence. Two such stages are subjective cognitive decline (SCD) and mild cognitive impairment (MCI) [8].

SCD is a self-reported persistent decline in cognitive functions with typical performance on standardized cognitive tests [9]. People with SCD have an increased risk of being positive for AD-specific biomarkers and, thus, of developing objective cognitive decline and progression to dementia [8, 10]. Individuals with MCI, in contrast, already exhibit noticeable cognitive deficits, often in memory, language, or executive function, but retain independence in daily life. This stage is clinically significant because MCI increases the likelihood of progressing to dementia [11, 12].

Several studies have assessed healthcare service utilization and costs across severity stages of AD dementia (e.g. review [13]), indicating increasing costs with more severe disease stages with a high percentage of informal care costs. Even though costs are highest in the most severe stages, there is an increasing number of studies, including pre-dementia stages, such as MCI [14–20], and few studies reporting SCD costs [21, 22]. These studies showed elevated costs in SCD and MCI. Evidence on cost differences across all stages from SCD to AD dementia (ADD) using the same cost components and perspectives is still scarce, as is evidence on associated potential cost drivers, such as genetic, clinical, or exogenous risk factors.

Our study aimed to assess the economic burden from the public payer and societal perspective across the entire Alzheimer's disease continuum compared to healthy controls. Based on previous studies that demonstrated higher costs at higher disease stages, we hypothesize that the costs in SCD and MCI are already elevated compared to those in healthy controls. Furthermore, we assessed if healthcare costs differ between patients with and those without the genetic risk factor APOE e4 or the biomarker Aß. Finally, we aimed to explore the association of costs and several other modifiable risk factors for AD. Both association analyses are exploratory without predefined hypotheses.

## Methods

### Study design

This cross-sectional analysis is based on data from the DZNE-Longitudinal Cognitive Impairment and Dementia Study (DELCODE), an observational longitudinal clinical cohort study conducted at ten German memory clinics of university hospitals collaborating with DZNE sites. The study's primary aim is to enhance knowledge about SCD with regard to cross-sectional features and longitudinal outcomes. Also, individuals with MCI, mild AD, relatives of patients with AD, and cognitively unimpaired controls are studied.

In total, 1,078 participants were enrolled in DELCODE. The respective study centers'local institutional review boards and ethical committees approved the study and the health economic add-on data collection. The DELCODE study protocol and the diagnostic criteria for group assignment are described in detail elsewhere [23].

### Participant groups

All patient groups (SCD, MCI, AD dementia) were recruited and assessed at the memory clinic study centres. The SCD group was characterized by a subjectively reported decline in cognitive function and everyday performance in the CERAD neuropsychological test battery (performance not worse than 1.5 standard deviations (SD) below demographically adjusted norms). The MCI group was defined by age, sex and education-adjusted performance below −1.5 SD on the delayed recall trial of the CERAD word-list episodic memory tests. The AD dementia patients had a clinical diagnosis of probable AD dementia according to the NIA-AA workgroup guidelines [24].

The control participants and first-degree relatives were recruited via newspaper advertisements. They had no objective cognitive impairment in cognitive tests, no history of neurological or psychiatric disease, and did not report a self-perceived cognitive decline. For the economic evaluation, the control group and the relatives were classified as healthy controls.

All participants or their representatives provided written informed consent. DELCODE was conducted in accordance with the Helsinki Declaration of 1975.

### Sample

All DELCODE participants underwent annual clinical follow-ups. The healthcare resource use assessment and the health economic evaluation were not planned initially but added to DELCODE as an add-on study during the ongoing patient follow-up visits between 2020 and 2022. Therefore, no economic data from the baseline data collection are available. Initially, it was planned to invite all DELCODE cohort participants to participate in the health economic data collection. However, not all study centres added the questionnaire to their assessment procedures, and not every patient or caregiver completed the health economic questionnaire. Therefore, data from six out of ten study centres and 375 out of 1,078 patients were available. The non-response rate was not recorded. Hence, we cannot compare the respondents to the non-responders in a drop-out analysis. For the health economic data collection, the Questionnaire for Health-Related Resource Use in an Elderly Population (FIMA) was used. Data collection for the health economic evaluation occurred between 2020 and 2022, which is equivalent to the follow-up visits 2 to 8. Most participants (81%) were in their follow-up visits 3 to 6. Of the 375 collected datasets, 51 participants were deleted due to duplicates or missing relevant variables. Thus, *n*=324 patients were included, of which (i) *n*=121 were classified as SCD, (ii) *n*=58 as MCI, (iii) *n*=31 as AD dementia, and (iv) *n*=114 as healthy controls belonging to either the control or the relative group. Group assignment was based on the results of the most recent annual neuropsychological tests at the time of the data collection. Hence, any progression in disease stages was reflected.

### Clinical and biomarker data assessment

All participants underwent annual clinical and neuropsychological testing and, if consented, Magnetic Resonance Imaging (MRI), biomaterial sampling and positron emission tomography (PET) screening.

### Health care resource data collection and costing

The FIMA questionnaire collects data on the frequency of the utilization of physician consultations, outpatient treatments (e.g., physical or speech therapy), hospital treatments, medications, and medical aids. Besides, hours of formal care (home care, day and night care) and informal care provided by close relatives were recorded. The recall period in the FIMA questionnaire varies between one week for medication, three months for physician treatment and formal and informal care and one year for hospital treatment, rehabilitation and medical aids. Cognitively healthy controls and participants with SCD and MCI completed the FIMA questionnaire independently (self-report), while AD dementia participants filled the questionnaire with the help of a relative.

Based on the reported resource utilization, the health care cost per patient was calculated by multiplication with published standardized unit costs [25]. Drug costs were taken from the Pharmaceutical Index of the Scientific Institute of the AOK, which is updated and published monthly [26]. By using the pharmaceutical registration number (PZN), the documented drugs were directly assigned to the pharmacy sales prices. All unit costs were inflated to 2023 (for 2020 0,5 %, for 2021 3,1 %, for 2022 6,9 %) using average annual inflation rates [27]. We calculated costs from the payer perspective (medical and formal care costs) and the societal perspective (adding informal care costs). All costs are displayed in Euros (€) for one year.

### Covariates

The following covariates were used to analyze the cost-driving factors across the AD continuum: the Charlson comorbidity index (CCI) [28], the Functional Activities Questionnaire (FAQ) [29], APOE [4] and Aß status [30]. The CCI reflects the comorbidity of patients based on 19 common diseases. The CCI score was calculated by assigning weighted points to a patient's comorbid conditions based on severity and cumulatively summarized with extra points for age. Participants were classified as APOE e4 positive if one or two alleles of APOE e4 were present. The Aß status was determined either via cerebrospinal fluid (CSF) Aß42/Aß40 ratio (cut-off value <= 0.08) or 18F-florbetaben (FBB; Neuraceq) PET scan (visual reading of scan, procedure described in [23]). Data on the Aß status were available for a subsample of 197 participants.

Furthermore, the following modifiable risk factors reported in the latest report of the Lancet Commission on dementia were included as dichotomous variables to conduct an exploratory analysis on their impact on the reported costs across the AD continuum: less education (years < 1 SD from the mean), hearing loss (self-reported difficulties), hypertension (ICD-10 diagnoses), smoking (ever smoked), obesity (body mass index>=30), depression (Geriatric Depression Scale >5 [31]), physical inactivity (PASE Score [32] <1 SD from the mean), diabetes (ICD-10 diagnoses E10. – E14.), excessive alcohol consumption (>168 g of ethanol per week), social isolation (Lubben Network Scale [33]< 1 SD from the mean), untreated vision loss (self-reported difficulties) and high LDL (ICD-10 diagnosis E78.0) [6].

### Statistical analysis

Participants'sociodemographic and clinical characteristics were demonstrated and compared across groups using descriptive statistics. Missing data on resource utilization were imputed using multiple imputations by chained equation. The differences in utilization of health care resources and costs between the groups were calculated using ANOVA (for continuous variables) and Chi^2^-test (for categorical variables). A generalized linear model (GLM) with gamma distribution and log link was used to assess the cost differences between healthy controls and SCD, MCI, and AD dementia. Since age and sex may influence costs [34] as well as CCI and FAQ, we adjusted for these factors to reduce their effects on costs. In a second exploratory analysis, we adjusted the model additionally for APOE status, Aß-status, or the presence of modifiable risk factors. Multicollinearity between all factors was tested beforehand, demonstrating poor correlations between factors (*r*^s^<0.3). However, age and CCI were moderately correlated only (*r*^s^=0.4.5). Since patients were recruited at six different study centres, we included a random effect to adjust for possible effects of the clusters (recruiting study centers) on the costs. Based on these models, adjusted mean costs from the payer and the societal perspective were generated and displayed using bar plots with error bars. A sensitivity analysis was carried out by truncating total costs to the 95% percentile if they had values above the 95% percentile. All statistical analyses were conducted in Stata SE version 17.

## Results

### Description of the study population

The description of participants is demonstrated in Table 1.

**Table 1:** Sociodemographic and clinical characteristics by cognitive impairment group

	controls	SCD	MCI	ADD	*p-value*
	*N*=114	*N*=121	*N*=58	*N*=31	
*Sociodemographics*					
Age, mean (SD)	73.4 (5.5)	73.7 (5.3)	76.8 (5.5)	79.3 (6.0)	<0.001
Sex (female), n (%)	67 (58.8%)	65 (53.7%)	27 (46.6%)	13 (41.9%)	0.26
Living community-dwelling, n (%)	110 (98.2%)	117 (98.3%)	56 (96.6%)	28 (90.3%)	0.09
Education (years), mean (SD)	15.3 (2.8)	14.5 (2.9)	14.3 (3.1)	14.1 (3.2)	0.05
*Clinical variables*					
Cognitive score (MMSE), mean (SD)	29.5 (1.0)	29.4 (0.9)	28.1 (2.1)	20.6 (5.1)	<0.001
FAQ score, mean (SD)	0.2 (0.7)	0.5 (1.5)	1.7 (3.3)	14.9 (9.8)	<0.001
Comorbidity (CCI), mean (SD)	3.7 (1.4)	3.6 (1.4)	3.8 (1.3)	4.5 (2.0)	0.02
APOE e4+, n (%)	28 (24.6%)	32 (26.4%)	24 (41.4%)	20 (64.5%)	<0.001
Aß+, n (%)	8 (15.7%)	23 (29.9%)	23 (54.8%)	24 (88.9%)	<0.001
*Risk factors*					
Sum risk factors, mean (SD)	2.0 (1.3)	2.3 (1.4)	2.6 (1.6)	2.7 (1.5)	0.01
Smoking, n (%)	56 (49.1%)	64 (52.9%)	29 (50.0%)	12 (38.7%)	0.57
Excessive alcohol consumption, n (%)	14 (12.3%)	10 (8.3%)	2 (3.4%)	3 (9.7%)	0.28
Obesity, n (%)	13 (11.4%)	22 (18.2%)	8 (13.8%)	2 (6.5%)	0.27
Less education, n (%)	7 (6.1%)	16 (13.2%)	9 (15.5%)	8 (25.8%)	0.02
Hearing loss, n (%)	12 (10.5%)	20 (16.5%)	12 (20.2%)	3 (9.7%)	0.24
Vision loss, n (%)	4 (3.5%)	5 (4.1%)	4 (6.9%)	2 (6.5%)	0.73
Depression, n (%)	3 (2.6%)	11 (9.1%)	7 (12.1%)	6 (19.4%)	0.01
Hypertension, n (%)	58 (50.9%)	69 (57.0%)	39 (67.2%)	20 (64.5%)	0.18
High cholesterol, n (%)	24 (21.1%)	32 (26.7%)	17 (29.3%)	12 (37.5%)	0.36
Diabetes, n (%)	3 (2.6%)	5 (4.1%)	2 (3.4%)	1 (3.2%)	0.94
Physical inactivity, n (%)	14 (12.3%)	17 (14.0%)	11 (19.0%)	12 (38.7%)	<0.001
Social isolation, n (%)	16 (14.0%)	11 (9.1%)	13 (22.4%)	4 (12.9%)	0.11

Data is presented as mean (SD) for continuous measures, and n (%) for categorical measures

Note that Aß status was only available for a subsample of patients (total sample size for which information on Aß-status was available: *n*=51 for controls, *n*=77 for SCD, *n*=42 for MCI and *n*=27 for ADD)

*Abbreviations* *SCD* subjective cognitive decline, *MCI* mild cognitive impairment, *ADD* Alzheimer’s disease dementia, *MMSE* Mini-Mental State Examination, *FAQ* Fuctional Activities Questionnaire, *CCI* Charlson Comorbidity Index, *APOE e4* apolipoprotein E4

Participants'age differed significantly across groups (*p*<0.001) with higher age in AD dementia and MCI than in SCD and controls. 52.9% were female with a lower, but not statistically significant proportion in higher disease stages. Almost all participants (97.2 %) were living community-dwelling. Mean education years and MMSE scores were significantly lower for cognitively impaired groups. Also, the CCI score, FAQ score, APOE e4-positive and Aß-positive status and the sum of existing risk factors were significantly higher in patients with SCI, MCI, and AD dementia than in healthy controls. These patients were also more likely to be depressed and physically inactive.

### Utilization of healthcare services and informal care across the AD continuum

The number of outpatient physician contacts increased from healthy controls (24.4 [18.7] visits per year) over SCD (32.0 [36.4]) and MCI (38.6 [64.0]) and dropped for AD dementia patients (27.5 [28.0]). A similar pattern was found for in-hospital treatment, therapies, and medical aids (see Table 2).

**Table 2:** Reported mean annual health care resource use by cognitive impairment group

	controls *N*=114	SCD *N*=121	MCI *N*=58	ADD *N*=31	*p*-value
*Medical care*					
Number of outpatient physician contacts, mean (SD)	24.4 (18.7)	32.0 (36.4)	38.6 (64.0)	27.5 (28.0)	0.12
* GP contacts*	6.4 (5.7)	7.9 (7.7)	8.3 (8.9)	7.7 (7.2)	0.31
* neurologist/psychiatrist contacts*	0.4 (1.4)	0.9 (2.7)	1.9 (3.5)	2.3 (3.1)	<0.001
* other specialist contacts*	17.6 (16.2)	23.1 (31.9)	28.4 (59.7)	17.4 (21.4)	0.20
In-hospital treatment, days, mean (SD)	3.6 (12.3)	3.3 (9.1)	3.9 (12.5)	1.7 (4.6)	0.82
Therapies, mean (SD)	12.1 (25.2)	17.1 (27.6)	18.8 (33.2)	13.8 (21.7)	0.37
Medical aids, mean (SD)	0.1 (0.5)	0.3 (0.7)	0.4 (0.9)	0.2 (0.5)	0.09
Medications, mean (SD)	3.3 (2.3)	4.2 (2.8)	4.5 (3.5)	5.8 (3.6)	<0.001
*Formal care*					
Ambulant care and support hours, mean (SD)	4.1 (26.0)	5.4 (28.2)	64.3 (383.5)	126.7 (364.8)	0.01
Residential care days, mean (SD)	0.0 (0.0)	0.3 (2.9)	0.0 (0.0)	5.7 (26.2)	0.01
*Informal care*					
Informal care and support hours, mean (SD)	8.8 (69.6)	52.7 (263.5)	287.2 (1223.4)	733.3 (1,657.5)	<0.001

*Abbreviations* *SCD* subjective cognitive decline, *MCI* mild cognitive impairment, *ADD* Alzheimer's disease dementia,* GP* general practitioner

The annual number of neurologist/psychiatrist visits (controls 0.4 [1.4], SCD 0.9 [2.7] vs ADD 2.3 [3.1]; *p*<0.001) and hours spent for formal ambulant (controls 4.1 [26.0], SCD 5.4 [28.2] vs ADD 126.7 [364.8]; *p*=0.005) and informal care (8.8 [69.6], SCD 52.7 [263.5] vs ADD 733.2 [1,657.5]; *p*<0.001) was significantly higher in SCD, MCI and ADD than in healthy controls. Residential care services (like day care) were almost exclusively used by patients with ADD (5.7 days per year [26.2]).

Compared to healthy controls, SCD patients visited outpatient physicians significantly more often (32.0 [36.4] vs 24.4 [18.7], *p*=0.048) and took significantly more medications (4.2 [2.8] vs 3.3 [2.3], *p*=0.013). When comparing all patients who were unimpaired in psychometrical tests (controls and SCD) vs patients with cognitive impairment (MCI and ADD), groups differed significantly in neurologist/psychiatrist contacts (*p*<0.001), medications (*p*=0.001), professional care and support (*p*=0.001) and informal care and support (*p*<0.001). Results of these subgroup comparisons are demonstrated in the Supplementary Tables 1 and 2.

### Differences in costs across the AD continuum

Unadjusted costs from the payer perspective increased from controls (4,346€ [6,780]) over SCD (5,532€ [7,363]), MCI (9,024€ [16,356]) and ADD (10,300€ [16,059]). From the societal perspective which includes informal care, this increase was even more pronounced (controls 4,653€ [7,221]; SCD 7,378€ [13,857]; MCI 19,078€ [47,728]; ADD 35,972€ [64,159]. Unadjusted costs are demonstrated in Table 3.

**Table 3:** Mean annual healthcare cost in Euro by cognitive impairment group

	Controls *N*=114	SCD *N*=121	MCI *N*=58	ADD *N*=31	*p*-value
*Medical care*					
Outpatient physician treatment, mean (SD)	1,358.82 (2,073.31)	1,738.58 (2,790.45)	2,216.19 (4,972.95)	1,518.60 (1,693.78)	0.36
* GP treatment*	192.50 (170.06)	236.87 (229.35)	248.11 (265.66)	232.11 (216.60)	0.31
* neurologist/psychiatrist treatment*	24.84 (96.50)	65.52 (192.61)	131.82 (250.00)	164.42 (216.57)	<0.001
* specialist treatment*	1,141.48 (2,048.41)	1,436.19 (2,630.74)	1,836.26 (4,765.46)	1,122.08 (1,481.50)	0.47
In-hospital treatment, mean (SD)	1,808.54 (5,734.77)	1,691.79 (4,118.63)	2,545.06 (8,478.84)	1,800.17 (5,053.67)	0.82
Therapies, mean (SD)	397.00 (1,010.10)	544.59 (859.94)	661.15 (1,333.78)	655.78 (1,092.80)	0.35
Medical aids, mean (SD)	66.91 (274.72)	173.82 (439.65)	202. 44 (614.21)	38.87 (125.69)	0.06
Medications, mean (SD)	589.86 (899.55)	1,188.72 (1,881.34)	1,435.35 (2,085.46)	1,646.90 (1,646.72)	<0.001
*Formal care*					
Ambulant care and support, mean (SD)	31.30 (196.75)	44.34 (236.97)	491.04 (2,856.23)	1,069.48 (2,799.60)	<0.001
Residential care, mean (SD)	0.00 (0.00)	16.79 (184.67)	0.00 (0.00)	361.69 (1,736.11)	0.01
*Informal care*					
informal care and support, mean (SD)	306.59 (2,436.95)	1,846.04 (9,223.35)	10,053.91 (42,831.00)	25,671.82 (58,030.45)	<0.001
*Total cost*					
Payers perspective, mean (SD)	4,346.35 (6,780.02)	5,531.66 (7,362.95)	9,024.33 (16,356.40)	10,299.91 (16,058.73)	0.01
Societal perspective, mean (SD)	4,652.94 (7,220.66)	7,377.70 (13,856.74)	19,078.24 (47,727.81)	35,971.73 (64,159.44)	<0.001

*Abbreviations* *SCD* subjective cognitive decline, *MCI* mild cognitive impairment, *ADD* Alzheimer's disease dementia, *GP* general practitioner

In the multivariate regression model, the adjusted costs, compared to healthy controls, were 26% (b=1.26, 95%CI [0.96–1.64], *p*=0.09), 85% (b=1.85, [1.30–2.63], *p*=0.001), and 36% (b=1.36, 0.70–2.64, *p*=0.37) higher from the payer's perspective in SCD, MCI, and AD, respectively (see Table 4). Corresponding adjusted mean healthcare costs per year were 4,754 € [3, 586-5 ,922€], 5,976 € [95%CI 4, 598-7 ,355€], 8,795 € [95%CI 6, 200-11 ,391€] and 6,454 € [2, 796-10 ,111€] for healthy controls, SCD, MCI and AD dementia, respectively (see Table 5).

**Table 4:** Multivariate association between cognitive impairment group and total payer and societal costs

	Payer Perspective (Medical+formal care)	Societal Perspective (Medical+formal+informal care)
	*exp b (SE) [95% CI]*	*exp b (SE) [95% CI]*
*AD continuum (ref. healthy controls)*		
* Subjective Cognitive Decline*	1.26 (0.17) [0.96–1.64]	1.52 (0.24) [1.11 - 2.08]**
* Mild Cognitive Impairment*	1.85 (0.33) [1.30–2.63]**	2.70 (0.60) [1.74 - 4.17]***
* Alzheimer's Disease dementia*	1.36 (0.46) [0.70–2.64]	4.07 (1.33) [2.14–7.73]***
*Demographical and clinical factors*		
Age	1.01 (0.01) [0.99–1.04]	1.03 (0.02) [1.00 - 1.06]
Sex (Ref. female)	1.16 (0.14) [0.91–1.47]	1.43 (0.20) [1.08 - 1.89]*
Functional impairment (FAQ)	1.01 (0.02) [0.98–1.05]	1.03 (0.02) [1.00–1.06]*
Comorbidity (CCI)	1.17 (0.06) [1.06–1.29]**	1.10 (0.07) [0.97 - 1.23]

The generalized linear model with gamma function and log link was adjusted for sex, age, FAQ and CCI.

*Abbreviations* *FAQ* Functional Activities Questionnaire, *CCI* Charlson Comorbidity Index, *exb b* the exponentiated value of the regression coefficient b, *SE* standard error, *CI* confidence interval

*p<0.05

**p<0.005

***p<0.001

**Table 5:** Adjusted mean annual healthcare cost in Euro by cognitive impairment group

	Controls	SCD	MCI	ADD
	*N*=114	*N*=121	*N*=58	*N*=31
*Total costs*				
Payers perspective	4,753.77 (595.82)	5,976.50 (703.53)	8,795.29 (1,324.16)	6453.75 (1,866.01)
Societal perspective	5,522.32 (871.43)	8,377.28 (1,208.35)	14,886.05 (2,735.75)	22,481.43 (6,371.49)

Costs were adjusted for sex, age, FAQ and CCI via generalized linear model with gamma function and log link

Data are presented as mean (SD)

*Abbreviations* *SCD* subjective cognitive decline, *MCI* mild cognitive impairment, *ADD* Alzheimer's disease dementia

*p<0.05

**p<0.005

***p<0.001

From a societal perspective that includes costs for informal care, adjusted costs were 52% (b=1.52, 95% CI[1.11–2.08], *p*=0.009) higher in SCD, 170% in MCI (b=2.70, [1.74–4.17], *p*<0.001) and 307% in AD (b=4.07, [2.14–7.73], *p*<0.001) as compared to healthy controls (see Table 4), with corresponding adjusted mean healthcare costs per year of 5,522€ [95%CI 3,814-7 ,230€], 8,377€ [6,009–10,746€)], 14,886€ [9,524-20 ,248€] and 22,481€ [9,994-34 ,969€] for controls, SCD, MCI and AD dementia, respectively (see Table 5).

The sensitivity analysis (truncated cost outliers) confirmed these results (see supplementary material). While age was not significantly associated with cost, comorbidities had a significant effect on costs from a payer perspective and sex and functional impairment from a societal perspective (see Table 4).

### Associated factors

Hypertension was associated with higher healthcare costs from both payer and societal perspectives. Vision loss and diabetes were also associated with higher costs from a payer perspective. In contrast, from a societal perspective, social isolation was associated with higher costs and lower education status with reduced costs (see Table 6).

**Table 6:** Multivariate association between cognitive impairment group and total societal costs including clinical and modifiable risk factors

	Payer Perspective (Medical+formal care)	Societal Perspective (Medical, formal, informal care)
	*exp b (SE) [95% CI]*	*exp b (SE) [95% CI]*
*AD continuum (ref. healthy controls)*		
* Subjective Cognitive Decline*	1.32 (0.18) [1.01–1.71]*	1.61 (0.24) [1.20–2.17]**
* Mild Cognitive Impairment*	1.82 (0.33) [1.28–2.59]**	2.57 (0.54) [1.70–3.89]***
* Alzheimer's Disease dementia*	1.54 (0.50) [0.82–2.91]	5.33 (1.63) [2.92–9.71]***
*Demographics*		
Age	1.03 (0.01) [1.00–1.06]	1.04 (0.02) [1.00–1.07]*
Sex (Ref. female)	1.06 (0.13) [0.83–1.35]	1.23 (0.17) [0.93–1.62]
*Clinical characteristics*		
APOE status (ref. non-carrier)	0.78 (0.11) [0.60–1.02]	0.68 (0.10) [0.51–0.91]*
Comorbidity (CCI)	1.09 (0.06) [0.98–1.20]	1.07 (0.06) [0.95–1.20]
Functional impairment (FAQ)	1.01 (0.02) [0.98–1.04]	1.02 (0.02) [0.99–1.05]
Modifiable risk factors		
Smoking	0.91 (0.11) [0.71–1.15]	0.84 (0.12) [0.63–1.12]
Excessive alcohol consumption	1.11 (0.24) [0.73–1.68]	1.03 (0.25) [0.63–1.67]
Obesity	1.01 (0.17) [0.72–1.41]	1.06 (0.21) [0.72–1.57]
Less education	0.73 (0.14) [0.50–1.06]	0.54 (0.11) [0.35–0.81]**
Hearing loss	1.22 (0.21) [0.87–1.71]	1.36 (0.27) [0.92–2.01]
Vision loss	2.08 (0.58) [1.20–3.60]*	1.67 (0.52) [0.91–3.09]
Depression	1.23 (0.26) [0.80–1.87]	1.15 (0.29) [0.70–1.90]
Hypertension	1.27 (0.15) [1.00–1.60]*	1.46 (0.21) [1.10–1.93]*
High cholesterol	0.79 (0.11) [0.60–1.04]	0.65 (0.10) [0.47–0.88]
Diabetes	2.25 (0.75) [1.17–4.33]*	2.32 (0.91) [1.07–5.02]
Physical inactivity	1.18 (0.20) [0.85–1.63]	1.10 (0.21) [0.75–1.60]
Social isolation	1.35 (0.23) [0.96–1.89]	1.58 (0.32) [1.06–2.35]*

The generalized linear model with gamma function and log link was adjusted for sex, age, CCI, APOE, and modifiable risk factors

*Abbreviations* *CCI* Charlson Comorbidity Index, *APOE* apolipoprotein E, *FAQ* Functional Activities Questionnaire, *exp b* the exponentiated value of the regression coefficient b, *SE* standard error, *CI* confidence interval

*p<0.05

**p<0.005

***p<0.001

**Fig. 1:**
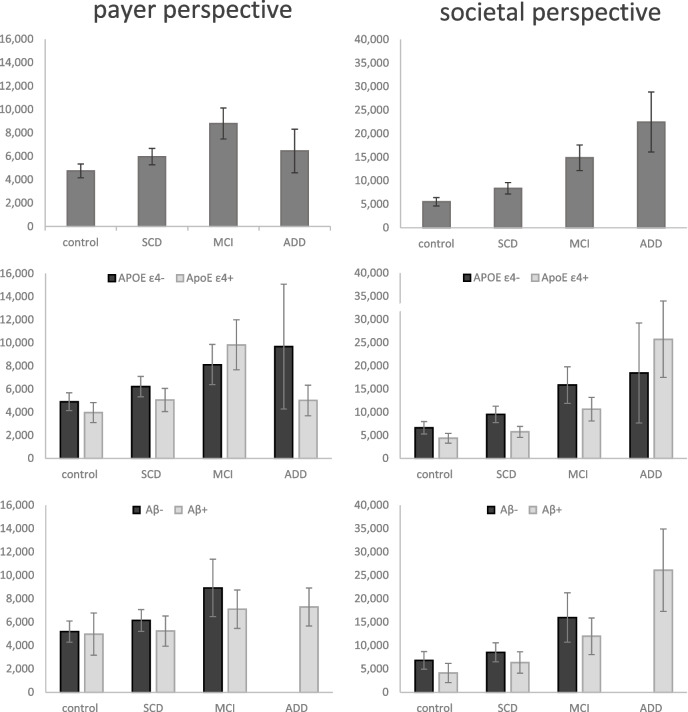
Adjusted mean annual cost from payer and societal perspective per disease stage. Costs are adjusted for age, sex, functional impairment and comorbidities. Additionally, subgroups for APOE and Aβ status are presented. Note that Aβ status was only available for a subsample (total sample size for which information on Aβ-status was available: *n*=51 for controls, *n*=77 for SCD, *n*=42 for MCI and *n*=27 for ADD)

After separating the sample according to the APOE and Aß status, we compared the costs for positive and negative patients in each group. We found that APOE e4-negative patients incurred higher costs than APOE e4-positive patients in the same group for all disease stages except for MCI, where costs from the payer perspective were slightly lower. Concerning Aß, patients without amyloid deposition had higher costs in all groups. Figure 1 demonstrates the adjusted mean costs from the payer and societal perspective across the AD continuum and for APOE and Aß subgroups.

## Discussion

This study assessed healthcare utilization and costs from the payer and the societal perspective across the AD continuum, ranging from SCD, MCI, and AD dementia, compared to healthy controls. Societal costs were significantly elevated across all stages: 52% higher in SCD (€8,377), 170% higher in MCI (€14,886), and 307% higher in AD dementia (€22,481) compared to controls (€5,522). Formal and informal care costs, along with medication expenses, rose with disease progression. In contrast, costs for physician treatments and medical aids were lower in AD dementia than in MCI and SCD. Hypertension was linked to higher costs, as were a positive APOE e4 or Aß status.

Prior studies confirm that healthcare costs increase across early AD stages [14, 15, 22]. Leibson et al. [14] reported rising costs from healthy controls to MCI and dementia for inpatient and outpatient treatment, though outpatient costs declined in prevalent dementia cases. Ton et al. [15] also reported increasing costs for MCI and different AD severity stages, demonstrating an increase in inpatient and care services but a steady decrease in physician consultations. One previous study reported costs ranging from 6,196€−7,104€ for SCD, 8,452€−9758€ for MCI and 9,783€−11,728€ for AD dementia from the payer perspective [22]. Our study aligns with these trends, showing progressively higher costs with disease severity, except for certain services like physician or hospital treatments, which decline in late stages. This reduced service utilization in AD dementia patients may reflect reduced mobility, dependence on caregivers or difficulties in expressing needs. Comparisons of absolute costs between studies are challenging due to different cost perspectives and diverse healthcare systems across countries. Nonetheless, our study adds further data on the actual costs of healthcare services.

A unique strength of our study is the inclusion of an SCD group besides cognitively healthy participants and patients with MCI, contributing to filling an important knowledge gap. Only one previous study compared healthcare service utilization for SCD to healthy controls [21], reporting fewer physician practice consultations but more hospital stays and formal care for patients with SCD, resulting in 60% increased costs. We found a similar pattern with an increased service utilization in SCD compared to healthy controls and elevated costs by 25% from a payer and by 50% from a societal perspective, confirming the previous study results. Although SCD patients show no objective impairments in neuropsychological tests, they might more frequently seek medical evaluations driven by concerns about their cognitive health, resulting in increased diagnostic testing [35]. Also, individuals with SCD often experience higher levels of anxiety and psychological distress [36], which may lead to elevated healthcare service utilization, as demonstrated in our study.

Even though informal care is a substantial cost factor in dementia and AD [37], accounting for 50 to 90% of the total cost [13], evidence on informal care and, thus, the societal perspective is still scarce in early AD stages, especially in SCD. Our study showed that informal care provision and, thus, informal care costs were already elevated in SCD compared to healthy controls and significantly increased further in the progression of cognitive impairment. This is an interesting finding as the definition of SCD and MCI states that individuals'performance in daily functions is not yet impaired. Still, our data showed that they required significantly more informal care than healthy controls, which may relate to subtle functional impairments in performing everyday tasks emerging already in the SCD stage [38]. However, the cost difference remains even after adjustment for functional disabilities. Hence, there are more factors to consider. Another factor may be mild behavioural impairment in preclinical and prodromal stages, which may contribute to the need for informal care [39]. Our findings agree with studies on the societal costs of MCI, showing a relevant need for informal care [16, 40] Further research on informal care provision across the AD continuum may help identify in which fields patients require informal care despite having no or little limitations in executing daily functions.

Being male and functional impairment were significantly associated with increased costs from a societal perspective, while CCI was significantly associated from a payer perspective. CCI mainly drives direct medical costs [41], while male sex and functional impairment have been shown to be linked with an increase in informal care [42, 43] and thus higher societal costs.

In addition, we observed that APOE e4-negative patients incurred higher costs compared to APOE e4-positive patients within the same group across most disease stages. These findings were not statistically significant, however it may be interesting to look into this trend as it appears counterintuitive. APOE e4 is known to enhance Aß accumulation in AD patients [44], trigger inflammation cascades [45], aggravate tau pathology and potentiate tau-mediated neurodegeneration, which is associated with accelerated neurodegeneration [3], possibly leading to higher needs for healthcare services. Besides, APOE4 has been linked to an elevated risk for cardiovascular diseases, but a reduced risk for various common types of cancers [46]. These antagonistic effects were reported to be linked with an overall increased mortality risk [47–49] for APOE e4 carriers. Further research is needed to elucidate how these opposing effects of APOE e4 influence healthcare utilization and cost patterns across the disease continuum. A possible explanation for the higher costs in controls and SCD suggests that APOE e4-positive individuals may exhibit greater resilience to remain in a lower stage of the disease due to protective factors, delaying progression and thereby reducing costs. Conversely, APOE e4-negative cases may require additional risk factors, e.g. comorbidities, to develop cognitive deficits, increasing the costs in the higher disease stages. It should be noted that this explanation is exploratory in nature and was not derived from an a priori hypothesis, highlighting the need for further research.

Looking at the participants'amyloid status, we found higher costs in Aß- individuals compared to individuals with Aß+ in the same group, possibly reflecting increased diagnostic efforts to clarify the cognitive symptoms or other comorbidities that may lead to the observed cognitive symptoms. Even though results are not significant, they are in line with the GERAS-US study [16] and a SveDem cohort study [20], which both investigated societal healthcare costs for MCI and AD dementia and the Amyloid status and found lower costs for Aß+ individuals with MCI and AD dementia.

Our analysis revealed that hypertension was significantly associated with increased costs across the AD continuum from both the payer and the societal perspective. Hypertension requires continuous monitoring, medication, and frequent medical visits, which can drive up costs [50]. Vision loss and diabetes were also linked to higher cost from the payer perspective, primarily driven by increased use of specialist and inpatient treatments, which is consistent with prior evidence [51, 52]. Interestingly, social isolation was associated with significantly higher costs from the societal, but not the payer perspective. Since informal care—mostly provided by relatives [53]—is the main cost driver from the societal perspective, one might assume that socially isolated individuals receive less informal care. However, our data suggest the opposite, raising questions about how social isolation and informal care interact in this population. Further research is warranted to explore this relationship. Additionally, lower education status reduced costs significantly from a societal perspective. Low education status is linked with lower health literacy and, thus, might limit the access and utilization of healthcare services for patients in all disease stages [54]. As this part of the analysis was explorative, further research would be needed to understand the impact of modifiable risk factors on costs alongside the AD continuum.

## Limitations

Limitations of the study include the relatively small sample size, affecting the statistical power of our analyses and the generalizability of the presented results, particularly in subgroup analyses. The cross-sectional design limits causal interpretations, and self-reported resource utilization may introduce recall bias. The control group was not a random population sample but searched for subjects feeling generally healthy and without cognitive impairments, potentially inflating cost discrepancies between controls and SCD. Furthermore, the control group of our sample also included relatives of AD patients, who may differ regarding their healthcare costs from a random population sample. However, this does not affect the absolute costs found for SCD, MCI, and AD dementia patients and the differences between these groups. The comparability between groups was limited since groups were selectively recruited rather than demonstrating a sample that progressed from healthy to SCD, MCI and, finally, AD. Not all individuals in the SCD- and MCI stages were amyloid-positive, meaning not all of them were likely on the AD pathway. Therefore, our results are not necessarily generalizable for an AD-only cohort in different cognitive impairment stages. Our subgroup analysis that includes the amyloid status already pointed out that the cost for amyloid-positive individuals seems lower than for amyloid-negative individuals. Hence, an all-cause SCD or MCI cohort is likely to reflect higher costs compared to an SCD or MCI cohort with amyloid-positivity. The resource consumption was collected in the follow-up visits between 2020 and 2022, which is for most individuals several years after recruitment. Patients who may have entered the study in a lower disease stage and progressed to a higher disease stage in a short time can be found in these respective advanced groups in our study. Thus, lower disease-stage groups no longer include these fast-progressing but relatively more stable patients. Costs per patient group would likely have differed if rapid progressors had remained in lower disease-stage groups. As data collection took place during the COVID pandemic, this might also have influenced patients'healthcare behaviour either by delaying non-time-critical treatments or by showing a catch-up effect depending on the time of data collection.

## Conclusion

Our findings highlight the substantial healthcare and societal costs that are already elevated in the early stages of the AD disease continuum. Costs increased consistently with disease severity, with informal care representing the primary cost driver even in pre-dementia stages. This underlines the importance of including early stages, such as SCD and MCI, in economic assessments of Alzheimer's disease, which is so far underrepresented in the literature. Most cost-effectiveness or budget impact analyses focus on the dementia stage, while the financial burden in earlier stages remains underrepresented. Our data provide a basis for refining such models by incorporating stage-specific cost estimates and accounting for heterogeneity related to biomarker status and modifiable risk factors.

Additionally, the observed cost differences across biomarker-defined subgroups, particularly those related to APOE, Aß status, or hypertension, highlight the complex interplay between biological and clinical factors in shaping healthcare utilization and costs. A better understanding of cost patterns across the AD continuum and associated factors can help to identify patient groups with higher care needs and costs to ameliorate information for health policy and decision-makers. Further research is urgently needed to explore the drivers of informal care provision in the early stages and to clarify the role of biological and modifiable risk factors in shaping healthcare costs over time.

## Supplementary Information

Supplementary Material 1

Supplementary Material 2

## Data Availability

The data analysed in this study are not publically available, but may be provided upon reasonable request. For details and contact, please see https://www.dzne.de/en/research/studies/clinical-studies/delcode/.

## Abbreviations

AD: Alzheimer's disease
ADD: Alzheimer's disease dementia
Aß: Amyloid-ß
b: Observed coefficient
CCI: Charlson comorbidity index
CI: Confidence interval
CSF: Cerebrospinal fluid
FIMA: Questionnaire for Health-Related Resource Use in an Elderly Population
GLM: Generalized linear model
MCI: Mild cognitive impairment
MMSE: Mini Mental State Examination
MRI: Magnetic Resonance Imaging
PET: Positron emission tomography
SCD: Subjective cognitive decline
SD: Standard deviation
