# Supplementary material for: The economic burden of subjective cognitive decline, mild cognitive impairment and Alzheimer's dementia: excess costs and associated clinical and risk factors
Source: Alzheimers Res Ther. 2025 Jun 26;17:142. doi: 10.1186/s13195-025-01785-9 (PMC12199487; doi:10.1186/s13195-025-01785-9)
Supplement: Supplementary file 1 — Supplementary Material 1 [file 13195_2025_1785_MOESM1_ESM.docx]

**Supplementary material**

**Supplementary table 1** Observed annual health care resource use for groups unimpaired in psychometrical testing

|  | **controls** | **SCD** | **p-value** |
| --- | --- | --- | --- |
|  | **N=114** | **N=121** |  |
| ***Medical care*** |  |  |  |
| Number of outpatient physician contacts, mean (SD) | 24.39 (18.65) | 31.97 (36.42) | **0.048** |
| *GP contacts* | 6.42 (5.67) | 7.90 (7.65) | 0.095 |
| *neurologist/psychiatrist contacts* | 0.35 (1.36) | 0.93 (2.72) | **0.044** |
| *other specialist contacts* | 17.61 (16.24) | 23.14 (31.88) | 0.098 |
| In-hospital treatment days, mean (SD) | 3.58 (12.32) | 3.25 (9.08) | 0.81 |
| Therapies, mean (SD) | 12.07 (25.21) | 17.12 (27.63) | 0.15 |
| Medical aids, mean (SD) | 0.13 (0.47) | 0.28 (0.73) | 0.073 |
| Medications, mean (SD) | 3.34 (2.34) | 4.18 (2.75) | **0.013** |
| ***Formal care*** |  |  |  |
| Ambulant care and support hours, mean (SD) | 4.13 (26.02) | 5.40 (28.19) | 0.72 |
| Residential care days, mean (SD) | 0.00 (0.00) | 0.26 (2.91) | 0.33 |
| ***Informal care*** |  |  |  |
| Informal care and support hours, mean (SD) | 8.76 (69.61) | 52.73 (263.45) | 0.086 |

**Supplementary Table 2** Observed health care resource use for participant group unimpaired in testing vs cognitively impaired participants

|  | **unimpaired in psycho-**  **metrical testing** | **Cognitively impaired** | **p-value** |
| --- | --- | --- | --- |
|  | **N=235** | **N=89** |  |
| ***Medical care*** |  |  |  |
| Number of outpatient physician contacts, mean (SD) | 28.29 (29.37) | 34.70 (54.29) | 0.17 |
| *GP contacts* | 7.18 (6.79) | 8.09 (8.29) | 0.31 |
| *neurologist/psychiatrist contacts* | 0.65 (2.19) | 2.02 (3.36) | **<0.001** |
| *other specialist contacts* | 20.46 (25.62) | 24.58 (49.89) | 0.33 |
| In-hospital treatment days, mean (SD) | 3.41 (10.75) | 3.15 (10.46) | 0.84 |
| Therapies, mean (SD) | 14.67 (26.55) | 17.03 (29.67) | 0.49 |
| Medical aids, mean (SD) | 0.21 (0.62) | 0.31 (0.79) | 0.21 |
| Medications, mean (SD) | 3.77 (2.59) | 4.95 (3.51) | **0.001** |
| ***Formal care*** |  |  |  |
| Ambulant care and support hours, mean (SD) | 4.78 (27.10) | 86.05 (376.17) | **0.001** |
| Residential care days, mean (SD) | 0.14 (2.09) | 1.98 (15.52) | 0.076 |
| ***Informal care*** |  |  |  |
| Informal care and support hours, mean (SD) | 31.40 (196.00) | 442.56 (1397.05) | **<0.001** |

**Supplementary Table 3** Sensitivity analysis of adjusted costs from payer and societal perspective

|  | **Payer Perspective**  (Medical+formal care) | **Societal Perspective**  (Medical, formal, informal care) |
| --- | --- | --- |
|  | *exp b (SE) [95% CI]* | *exp b (SE)* |
| ***AD continuum*** *(ref. healthy controls)* |  |  |
| Subjective Cognitive Decline | **1.36 (0.17) [1.08 – 1.73]*** | **1.42 (0.20) [1.08-1.87]*** |
| Mild Cognitive Impairment | **1.78 (0.27) [1.32 – 2.40]***** | **3.11 (0.57) [2.17-4.47]***** |
| Alzheimer's Disease Dementia | 1.55 (0.43) [0.89 – 2.67] | **8.51 (2.57) [4.71-15.39]***** |
| ***Demographical and clinical factors*** |  |  |
| Age | **1.03 (0.01) [1.01 – 1.05]*** | 1.00 (0.01) [0.98-1.03] |
| Sex (Ref. female) | 1.03 (0.11) [0.84 – 1.27] | **1.27 (0.15) [1.00-1.61]*** |
| Functional impairment (FAQ) | 1.02 (0.01) [0.99 – 1.05] | 0.98 (0.02) [0.95-1.01] |
| Comorbidity (CCI) | 1.00 (0.05) [0.91-1.10] | **1.18 (0.06) [1.06-1.31]*** |

*For each group costs were truncated at the 95^th^ percentile. The generalized linear model with gamma function and log link was adjusted for sex, age, FAQ and CCI. Abbreviations:* FAQ Functional Activities Questionnaire; *CCI, Charlson Comorbidity Index; exp b, the exponentiated value of the regression coefficient b, SE, standard error; CI, confidence interval.*

* p<0.05 ** p<0.005 *** p<0.001
