# Supplementary material for: The economic burden of subjective cognitive decline, mild cognitive impairment and Alzheimer's dementia: excess costs and associated clinical and risk factors
Source: Alzheimers Res Ther. 2025 Jun 26;17:142. doi: 10.1186/s13195-025-01785-9 (PMC12199487; doi:10.1186/s13195-025-01785-9)
Supplement: Supplementary file 2 — Supplementary Material 2 [file 13195_2025_1785_MOESM2_ESM.docx]

|  | | | Item No. | Recommendation | | Page  No. | Relevant text from manuscript |
| --- | --- | --- | --- | --- | --- | --- | --- |
| **Title and abstract** | | | 1 | (*a*) Indicate the study’s design with a commonly used term in the title or the abstract | | 3 | The German DELCODE cohort study assessed clinical data, healthcare resource use, and informal care provision. |
|  |  |  |  | (*b*) Provide in the abstract an informative and balanced summary of what was done and what was found | | 3 | Costs were calculated from payer and societal perspectives using standardized unit costs, and multivariate regression analyses identified cost-associated factors. From a payer perspective, costs were elevated by 26% for SCD (…), 85% for MCI (…) and 36% for AD (…) compared to controls (…). Societal costs were elevated by 52% for SCD (…), 170% for MCI (…) and 307% for AD (…) compared to controls (…). APOE ε4 negative patients showed higher costs compared to APOE ε4 positive patients. Hypertension was associated with higher costs |
| Introduction | | | | | | |  |
| Background/rationale | | | 2 | Explain the scientific background and rationale for the investigation being reported | | 5 | Evidence on cost differences across all stages from SCD to AD dementia (ADD) using the same cost components and perspectives is still scarce, as is evidence on associated potential cost drivers, such as genetic, clinical, or exogenous risk factors. |
| Objectives | | | 3 | State specific objectives, including any prespecified hypotheses | | 5-6 | Our study aimed to assess the economic burden from the public payer and societal perspective across the entire Alzheimer's disease continuum compared to healthy controls. Based on previous studies that demonstrated higher costs at higher disease stages, we hypothesize that the costs in SCD and MCI are already elevated compared to those in healthy controls. Furthermore, we assessed if healthcare costs differ between patients with and those without the genetic risk factor APOE ε4 or the biomarker Aβ. Finally, we aimed to explore the association of costs and several other modifiable risk factors for AD. Both association analyses are exploratory without predefined hypotheses. |
| Methods | | | | | | |  |
| Study design | | | 4 | Present key elements of study design early in the paper | | 6 | observational longitudinal clinical cohort study |
| Setting | | | 5 | Describe the setting, locations, and relevant dates, including periods of recruitment, exposure, follow-up, and data collection | | 6 | (…) conducted at ten German memory clinics of university medicines collaborating with DZNE sites; Data collection for the health economic evaluation occurred between 2020 and 2022, which is equivalent to the follow-up visits 2 to 8. Most participants (81%) were in their follow-up visits 3 to 6. |
| Participants | | | 6 | (*a*) *Cohort study*—Give the eligibility criteria, and the sources and methods of selection of participants. Describe methods of follow-up | | 6-7 | All patient groups (SCD, MCI, AD dementia) were recruited and assessed at the memory clinic study centres. The SCD group was characterized by a subjectively reported decline in cognitive function and everyday performance in the CERAD neuropsychological test battery (…). The MCI group was defined by age, sex and education-adjusted performance below -1.5 SD on the delayed recall trial of the CERAD word-list episodic memory tests. The AD dementia patients had a clinical diagnosis of probable AD dementia according to the NIA-AA workgroup guidelines (24).  The control participants and first-degree relatives were recruited via newspaper advertisements. They had no objective cognitive impairment in cognitive tests, no history of neurological or psychiatric disease, and did not report a self-perceived cognitive decline. For the economic evaluation, the control group and the relatives were classified as healthy controls. (….) All DELCODE participants underwent annual clinical follow-ups. (…) Group assignment was based on the results of the most recent annual neuropsychological tests at the time of the data collection. Hence, any progression in disease stages was reflected. |
|  |  |  |  | (*b*) *Cohort study*—For matched studies, give matching criteria and number of exposed and unexposed | | N/A | (Not applicable since no matching of healthy controls was done.) |
| Variables | | | 7 | Clearly define all outcomes, exposures, predictors, potential confounders, and effect modifiers. Give diagnostic criteria, if applicable | | 8-10 | Based on the reported resource utilization, the health care cost per patient was calculated by multiplication with published standardized unit costs (25). Drug costs were taken from the Pharmaceutical Index of the Scientific Institute of the AOK, which is updated and published monthly (26). By using the pharmaceutical registration number (PZN), the documented drugs were directly assigned to the pharmacy sales prices. All unit costs were inflated to 2023 (for 2020 0,5 %, for 2021 3,1 %, for 2022 6,9 %) using average annual inflation rates (27). We calculated costs from the payer perspective (medical and formal care costs) and the societal perspective (adding informal care costs). All costs are displayed in Euros (€) for one year.  The following covariates were used to analyze the cost-driving factors across the AD continuum: the Charlson comorbidity index (CCI) (28), the Functional Activities Questionnaire (FAQ) (29) APOE (4) and Aβ status (30). The CCI reflects the comorbidity of patients based on 19 common diseases. The CCI score was calculated by assigning weighted points to a patient's comorbid conditions based on severity and cumulatively summarized with extra points for age. Participants were classified as APOE ε4 positive if one or two alleles of APOE ε4 were present. The Aβ status was determined either via cerebrospinal fluid (CSF) Aβ42/ Aβ40 ratio (cut-off value <= 0.08) or 18F‐florbetaben (FBB; Neuraceq) PET scan (visual reading of scan, procedure described in (23)). Data on the Aβ status were available for a subsample of 197 participants.  Furthermore, the following modifiable risk factors reported in the latest report of the Lancet Commission on dementia were included as dichotomous variables to examine their influence on the reported costs across the AD continuum: less education (years < 1 SD from the mean), hearing loss (self-reported difficulties), hypertension (ICD-10 diagnoses), smoking (ever smoked), obesity (body mass index≥30), depression (Geriatric Depression Scale >5 (31)), physical inactivity (PASE Score (32) <1 SD from the mean), diabetes (ICD-10 diagnoses E10. – E14.), excessive alcohol consumption (>168 g of ethanol per week), social isolation (Lubben Network Scale (33)< 1 SD from the mean), untreated vision loss (self-reported difficulties) and high LDL (ICD-10 diagnosis E78.0) (6). |
| Data sources/ measurement | | | 8* | For each variable of interest, give sources of data and details of methods of assessment (measurement). Describe comparability of assessment methods if there is more than one group | | *8* | All participants underwent annual clinical and neuropsychological testing and, if consented, Magnetic Resonance Imaging (MRI), biomaterial sampling and positron emission tomography (PET) screening. (…) Cognitive healthy controls and participants with SCD and MCI completed the FIMA questionnaire independently (self-report), while AD dementia participants filled the questionnaire with the help of a relative. |
| Bias | | | 9 | Describe any efforts to address potential sources of bias | | 8-10 | We addressed possible biases by taking different perspectives (payer and society), by using models to adjust the reported costs for several confounders and by applying sensitivity analyses to check if cost outliers had an effect on the results. |
| Study size | | | 10 | Explain how the study size was arrived at | | 7-8 | All DELCODE participants underwent annual clinical follow-ups. The healthcare resource use assessment and the health economic evaluation were not planned initially but added to DELCODE as an add-on study during the ongoing patient follow-up visits between 2020 and 2022. Therefore, no economic data from the baseline data collection are available. Initially, it was planned to invite all DELCODE cohort participants to participate in the health economic data collection. However, not all study centres added the questionnaire to their assessment procedures, and not every patient or caregiver completed the health economic questionnaire. Therefore, data from six out of ten study centres and 375 out of 1,011 patients were available. (…) Of the 375 collected datasets, 51 participants were deleted due to duplicates or missing relevant variables. |
| Quantitative variables | | 11 | | Explain how quantitative variables were handled in the analyses. If applicable, describe which groupings were chosen and why | | 8-10 | see information in point 7 |
| Statistical methods | | 12 | | (*a*) Describe all statistical methods, including those used to control for confounding | | 10 | Participants' sociodemographic and clinical characteristics were demonstrated and compared across groups using descriptive statistics. (…) The differences in utilization of health care resources and costs between the groups were calculated using ANOVA (for continuous variables) and Chi²-test (for categorical variables). A generalized linear model (GLM) with gamma distribution and log link was used to assess the cost differences between healthy controls and SCD, MCI, and AD dementia. Since age and sex influence costs (34) as well as CCI and FAQ, we adjusted for these factors to reduce their effects on costs. In a second exploratory analysis, we adjusted the model additionally for APOE status, Aβ-status, or the presence of modifiable risk factors. (…) Based on these models, adjusted mean costs from the payer and the societal perspective were generated and displayed using bar plots with error bars. |
|  |  |  |  | (*b*) Describe any methods used to examine subgroups and interactions | | 10 | Multicollinearity between all factors was tested beforehand, demonstrating poor correlations between factors (rs<0.3). However, age and CCI were moderately correlated only (rs=0.4.5). Since patients were recruited at six different study centres, we included a random effect to adjust for possible effects of the clusters (recruiting study centers) on the costs. |
|  |  |  |  | (*c*) Explain how missing data were addressed | | 10 | Missing data on resource utilization were imputed using multiple imputations by chained equation. |
|  |  |  |  | (*d*) *Cohort study*—If applicable, explain how loss to follow-up was addressed | | N/A | N/A; Since data from just one point in time were used, no losses to follow-up were applicable. |
|  |  |  |  | (*e*) Describe any sensitivity analyses | | 10 | A sensitivity analysis was carried out by truncating total costs to the 95% percentile if they had values above the 95% percentile. |
| Results | | | | | | | |
| Participants | | 13* | | (a) Report numbers of individuals at each stage of study—eg numbers potentially eligible, examined for eligibility, confirmed eligible, included in the study, completing follow-up, and analysed | | 6-7 | In total, 1,011 participants were enrolled in DELCODE. Initially, it was planned to invite all DELCODE cohort participants to participate in the health economic data collection. However, not all study centres added the questionnaire to their assessment procedures, and not every patient or caregiver completed the health economic questionnaire. Therefore, data from six out of ten study centres and 375 out of 1,011 patients were available. (…) Of the 375 collected datasets, 51 participants were deleted due to duplicates or missing relevant variables. |
|  |  |  |  | (b) Give reasons for non-participation at each stage | | 7 | (…) the non-response rate was not recorded. |
|  |  |  |  | (c) Consider use of a flow diagram | | N/A | N/A |
| Descriptive data | | 14* | | (a) Give characteristics of study participants (eg demographic, clinical, social) and information on exposures and potential confounders | | 11 | see Table 1 |
|  |  |  |  | (b) Indicate number of participants with missing data for each variable of interest | | N/A | N/A, since missing values were imputed |
|  |  |  |  | (c) *Cohort study*—Summarise follow-up time (eg, average and total amount) | | N/A | N/A, since only one point in time was used |
| Outcome data | | 15* | | *Cohort study*—Report numbers of outcome events or summary measures over time | | *N/A* | N/A, since only one point in time was used |
| Main results | | 16 | | (*a*) Give unadjusted estimates and, if applicable, confounder-adjusted estimates and their precision (eg, 95% confidence interval). Make clear which confounders were adjusted for and why they were included | | 14-16 | unadjusted results: Table 3; adjusted results: Table 4,5,6  confounders for Table 4 and 5: Since age and sex influence healthcare costs (34) as well as CCI and FAQ, we adjusted for these factors to reduce their effects on costs.  confounders for table 6: the following modifiable risk factors reported in the latest report of the Lancet Commission on dementia were included as dichotomous variables to conduct an exploratory analysis on their impact on the reported costs across the AD continuum: (…) |
|  |  |  |  | (*b*) Report category boundaries when continuous variables were categorized | | 9-10 | Participants were classified as APOE ε4 positive if one or two alleles of APOE ε4 were present. The Aβ status was determined (…) via cerebrospinal fluid (CSF) Aβ42/ Aβ40 ratio (cut-off value <= 0.08). (…) Modifiable risk factors(…): less education (years < 1 SD from the mean), (…), obesity (body mass index≥30), depression (Geriatric Depression Scale >5 (31)), physical inactivity (PASE Score (32) <1 SD from the mean), (…), excessive alcohol consumption (>168 g of ethanol per week), social isolation (Lubben Network Scale (33) < 1 SD from the mean) (…) |
|  |  |  |  | (*c*) If relevant, consider translating estimates of relative risk into absolute risk for a meaningful time period | | N/A | N/A |
| Other analyses | 17 | | Report other analyses done—eg analyses of subgroups and interactions, and sensitivity analyses | | 15 and Supplemetary | | The sensitivity analysis (truncated cost outliers) confirmed these results (see supplementary material). |
| Discussion | | | | | | | |
| Key results | 18 | | Summarise key results with reference to study objectives | | 18 | | This study assessed healthcare utilization and costs from the payer and the societal perspective across the AD continuum, ranging from SCD, MCI, and AD dementia, compared to healthy controls. Societal costs were significantly elevated across all stages: 52% higher in SCD (€8,377), 170% higher in MCI (€14,886), and 307% higher in AD dementia (€22,481) compared to controls (€5,522). Formal and informal care costs, along with medication expenses, rose with disease progression. In contrast, costs for physician treatments and medical aids were lower in AD dementia than in MCI and SCD. Hypertension was linked to higher costs, as were a positive ApoE ε4 or Aβ status. |
| Limitations | 19 | | Discuss limitations of the study, taking into account sources of potential bias or imprecision. Discuss both direction and magnitude of any potential bias | | 22-23 | | Limitations of the study include the relatively small sample size, affecting the statistical power of our analyses and the generalizability of the presented results, particularly in subgroup analyses. The cross-sectional design limits causal interpretations, and self-reported resource utilization may introduce recall bias. The control group was not a random population sample but searched for subjects feeling generally healthy and without cognitive impairments, potentially inflating cost discrepancies between controls and SCD. Furthermore, the control group of our sample also included relatives of AD patients, who may differ regarding their healthcare costs from a random population sample. However, this does not affect the absolute costs found for SCD, MCI, and AD dementia patients and the differences between these groups. The comparison between groups was limited since groups were selectively recruited rather than demonstrating a sample that progressed from healthy to SCD, MCI and, finally, AD. Not all individuals in the SCD- and MCI stages were amyloid-positive, meaning not all of them were likely on the AD pathway. Therefore, our results are not necessarily generalizable for an AD-only cohort in different cognitive impairment stages. Our subgroup analysis that includes the amyloid status already pointed out that the cost for amyloid-positive individuals seems lower than for amyloid-negative individuals. Hence, an all-cause SCD or MCI cohort is likely to reflect higher costs compared to an SCD or MCI cohort with amyloid-positivity. The resource consumption was collected in the follow-up visits between 2020 and 2022, which is for most individuals several years after recruitment. Patients who may have entered the study in a lower disease stage and progressed to a higher disease stage in a short time can be found in these respective advanced groups in our study. Thus, lower disease-stage groups no longer include these fast-progressing but relatively more stable patients. Costs per patient group would likely have differed if rapid progressors had remained in lower disease-stage groups. As data collection took place during the COVID pandemic, this might also have influenced patients' healthcare behaviour either by delaying non-time-critical treatments or by showing a catch-up effect depending on the time of data collection. |
| Interpretation | 20 | | Give a cautious overall interpretation of results considering objectives, limitations, multiplicity of analyses, results from similar studies, and other relevant evidence | | 18-22 | | Prior studies confirm that healthcare costs increase across early AD stages. (…)Our study aligns with these trends, showing progressively higher costs with disease severity (…)A unique strength of our study is the inclusion of an SCD group besides cognitively healthy participants and patients with MCI, contributing to filling an important knowledge gap. Only one previous study compared healthcare service utilization for SCD to healthy controls (21), reporting fewer physician practice consultations but more hospital stays and formal care for patients with SCD, resulting in 60% increased costs. We found a similar pattern (…)Even though informal care is a substantial cost factor in dementia and AD (37), accounting for 50 to 90% of the total cost (13), evidence on informal care and, thus, the societal perspective is still scarce in early AD stages, especially in SCD. Our study showed that informal care provision and, thus, informal care costs were already elevated in SCD compared to healthy controls and significantly increased further in the progression of cognitive impairment. (…)In addition, we observed that APOE ε4-negative patients incurred higher costs compared to APOE ε4-positive patients within the same group across most disease stages. These findings were not statistically significant, however it may be interesting to look into this trend as it appears counterintuitive. APOE ε4 is known to enhance Aβ accumulation in AD patients (44), trigger inflammation cascades (45), aggravate tau pathology and potentiate tau-mediated neurodegeneration, which is associated with accelerated neurodegeneration (3), possibly leading to higher needs for healthcare services. Looking at the participants' amyloid status, we found higher costs in Aβ- individuals compared to individuals with Aβ+ in the same group, possibly reflecting increased diagnostic efforts to clarify the cognitive symptoms or other comorbidities that may lead to the observed cognitive symptoms. Even though results are not significant, they are in line with the GERAS-US study (16) and a SveDem cohort study (20) |
| Generalisability | 21 | | Discuss the generalisability (external validity) of the study results | | 22-23 | | (…) relatively small sample size, affecting the statistical power of our analyses and the generalizability of the presented results (…)The control group was not a random population sample but searched for subjects feeling generally healthy and without cognitive impairments, potentially inflating cost discrepancies between controls and SCD. (…)The comparability between groups was limited since groups were selectively recruited rather than demonstrating a sample that progressed from healthy to SCD, MCI and, finally, AD. Not all individuals in the SCD- and MCI stages were amyloid-positive, meaning not all of them were likely on the AD pathway. Therefore, our results are not necessarily generalizable for an AD-only cohort in different cognitive impairment stages. Our subgroup analysis that includes the amyloid status already pointed out that the cost for amyloid-positive individuals seems lower than for amyloid-negative individuals. Hence, an all-cause SCD or MCI cohort is likely to reflect higher costs compared to an SCD or MCI cohort with amyloid-positivity. |
| Other information | | |  | | | | |
| Funding | 22 | | Give the source of funding and the role of the funders for the present study and, if applicable, for the original study on which the present article is based | | 29 | | This research did not receive any specific grant from funding agencies in the public, commercial, or not-for-profit sectors. |
